# Maternal *β*-Hemolytic Streptococcal Pharyngeal Exposure and Colonization in Pregnancy

**DOI:** 10.1155/2014/639141

**Published:** 2014-08-20

**Authors:** Giv Heidari-Bateni, Anoop K. Brar, Matthew Hall, Trupti Hathcock, Deirdre Epstein, Lisa S. Goessling, Madeleine W. Cunningham, Pirooz Eghtesady

**Affiliations:** ^1^Division of Pediatric Cardiothoracic Surgery, Washington University School of Medicine, St. Louis, MO 63110, USA; ^2^Children's Hospital Association, 6803 West 64th Street, Overland Park, KS 66202, USA; ^3^Department of Microbiology and Immunology, The University of Oklahoma Health Sciences Center, 940 Stanton L. Young Boulevard, BMSB 1053, Oklahoma City, OK 73104, USA

## Abstract

*Objectives*. To report the pharyngeal colonization rate of *β*-hemolytic streptococci and changes in the value of antistreptolysin O (ASO) and anti-DNase B serology titers during pregnancy. *Methods*. Healthy pregnant women were recruited and blood was drawn in each trimester. The upper limit of normal (ULN) values for ASO and anti-DNase B was calculated for each trimester. Throat swabs were collected for culture and positive cultures were further assessed for the identification of serogroup of the isolated *β*-hemolytic streptococcus. *Results*. Out of a total of 126 pregnant women, 34.1% had positive throat cultures. Group C and group G strains were isolated in 18.2% of throat cultures while group F was detected in 13.5% of cases. The rate of colonization with GAS was 1.6%. There was an overall drop in ASO titer during pregnancy while anti-DNase B titers remained relatively unchanged. ULN values of 164^IU^, 157^IU^, and 156^IU^ were calculated for ASO at the first, second, and third trimesters, respectively. Based on the ULN values, 28.6% of patients had recent streptococcal exposure. *Conclusions*. These results show that pregnant women act as a reservoir for spreading potentially immunogenic (groups C and G) and disease producing (group F) virulent strains of streptococci.

## 1. Introduction

Pregnancy is associated with physiological changes in the respiratory system [1]. Prior studies have suggested that pregnant women, as a result of modulations in the immune system, are more susceptible to upper respiratory tract infections [1, 2]. There are also concerns about the possible effect of respiratory infections during pregnancy on pregnancy outcomes [3–5]. Our group has previously suggested that maternal exposure to rheumatogenic groups of *β*-hemolytic streptococcus may play a role in pathogenesis of congenital heart disease in the unborn fetus [6], potentially mediated by streptococcal-induced anti-cardiac myosin antibodies [7]. There have been relatively few studies that have specifically studied *β*-hemolytic exposure during pregnancy. The point prevalence of *β*-hemolytic streptococcal pharyngeal colonization in the last trimester of pregnancy has been recently reported to be 8% in a small group of pregnant women in United Kingdom [8]. The pregnancy incidence rate, however, has not yet been studied and is anticipated to be much more than the reported point prevalence. For the general population, the two-year incidence rate has been reported to be 19.4% [9].

Although it is not well defined whether eradication of the asymptomatic carrier state is beneficial to the carrier hosts and their close contacts, it has been reported that the presence of carrier state might reduce the efficacy of penicillin G on eradicating group A streptococcal (GAS) pharyngitis [10, 11] and may be a source for both symptomatic or asymptomatic spread of GAS [12–14]. Furthermore, some strains of Lancefield groups C and G *β*-hemolytic streptococci (*Streptococcus dysgalactiae* subspecies* equisimilis*) possess common antigenic features with GAS [15–17] and can cause infections similar to GAS [8]. They are similarly attributed to induce rheumatogenic outcomes [16, 17].

The purpose of this study was to determine the rate of throat colonization and exposure to *β*-hemolytic streptococcus in pregnant women. We also defined the upper limit of normal (ULN) values of ASO and anti-DNase B titers in pregnant women.

## 2. Materials and Methods

### 2.1. Subjects

Pregnant subjects (>18 years of age) were recruited in the first trimester of the pregnancy and participated in a total of 3 visits. The first visit was at <18 weeks of gestation, the second between 20 and 30 weeks, and the third between 30 and 38 weeks. Subjects who received a blood transfusion or immunoglobulin infusion within 6 months of the pregnancy were excluded from the study. Only data from those subjects who completed all 3 visits are reported here.

This study was initiated in Cincinnati and moved to St. Louis upon transition of the principal investigator to Washington University, St. Louis, MO. The Institutional Review Boards at the Washington University School of Medicine, St. Louis, Missouri, and Cincinnati Children's Hospital Medical Center, Cincinnati, Ohio, approved this study. Written informed consent was received prior to inclusion in the study.

### 2.2. Questionnaires and Streptococcus Culture

All subjects completed a short questionnaire (see supplemental file in Supplementary Material available online at http://dx.doi.org/10.1155/2014/639141) at the first visit. This questionnaire was used to determine the mothers' history of antecedent *β*-hemolytic streptococcal infection.

Two throat swabs were collected at each visit and were cultured and grouped in accordance with Centers for Disease Control and Prevention protocols for Lancefield streptococcal identification (methods as described at http://www.cdc.gov/ncidod/biotech/strep/protocols.htm. date accessed: 12/26/13).

The incidence of *β*-hemolytic streptococcus throat colonization in pregnancy was defined as the number of subjects with at least one time positive throat culture divided by the total number of participants. The point prevalence of streptococcus throat colonization for each separate trimester was defined as the number of positive throat cultures divided by the total number of samples collected in that particular trimester. Carriers for *β*-hemolytic streptococcus were identified as subjects for whom the organism was isolated from at least two consecutive throat cultures while they were not symptomatic [18, 19], and there was no increase in ASO or anti-DNase B antibody titers [19].

### 2.3. ASO and Anti-DNase B Antibody Titers

Blood samples were collected from the subjects at each of the three visits to measure titers of ASO and anti-DNase B. Measurements of ASO and anti-DNase B titers were conducted by Quest Diagnostics Incorporated using quantitative immunoturbidimetric assays (for ASO) and by either Wampole Streptonase kit or immunonephelometric technique (for anti-DNase B). In order to identify the prevalence of recent exposure to streptococcus in the pregnant population, we utilized UNL 20% values of ASO and anti-DNase B titers (measured by Wampole kit) as described in the data analysis section. For anti-DNase B titers measured by immunonephelometric technique, due to a limitation in sample size, the ULN value presented by the reference laboratory was used. Subjects with a titer more than the ULN 20% value of either ASO or anti-DNase B (or a titer more than laboratory reference for anti-DNase B measured by the immunonephelometric technique) were considered as those with recent exposure to *β*-hemolytic streptococcus.

### 2.4. Data Analysis

Values of ASO and anti-DNase B titers were compared between trimesters using paired sample *t*-test. Changes in serology titers between pregnancy trimesters were measured for each subject; the median and the interquartile range for these changes within the study population were reported. Correlation between values of ASO and anti DNase-B antibodies was assessed for each pregnancy trimester.

Subjects were assigned into positive or negative groups based on throat culture results and the frequency of positive *β*-hemolytic streptococcus culture result in each trimester. The incidence of streptococcal colonization in pregnancy was measured as described earlier.

A ULN of 20% was considered as the 80th percentile of cumulative frequencies of unexposed population titers [20, 21]. Subjects with a titer more than the ULN 20% values were considered as those with recent exposure. In order to calculate the ULN value, subjects with evidence of infection with streptococcus from six months prior to blood draw were excluded as previously described [20, 21]. For this aim, we excluded subjects who showed at least a two-time increase in the level of ASO or anti-DNase B during pregnancy. Also, we excluded subjects who were prescribed an antibiotic for a sore throat from the time point of 3 months before the pregnancy and the time of blood draw.

Demographic data and responses to the questionnaire were compared between exposed and unexposed individuals, as well as those with positive and negative cultures using Fisher's exact test and Mann-Whitney *U* test to compare categorical and numerical data, respectively. A *P* value < 0.05 was considered as statistically significant when comparing the demographic data. The Bonferroni correction for *P* value (*P* = 0.0025) was used to reduce the chances of obtaining false-positive results when comparing the questionnaire-based data.

## 3. Results

A total of 126 subjects (from a total of 150 recruitments) presented for three visits between 18 and 38 weeks of pregnancy. Their demographic characteristics are shown in Table 1. A total of 7.9% of subjects were smokers.

**Figure 1:**
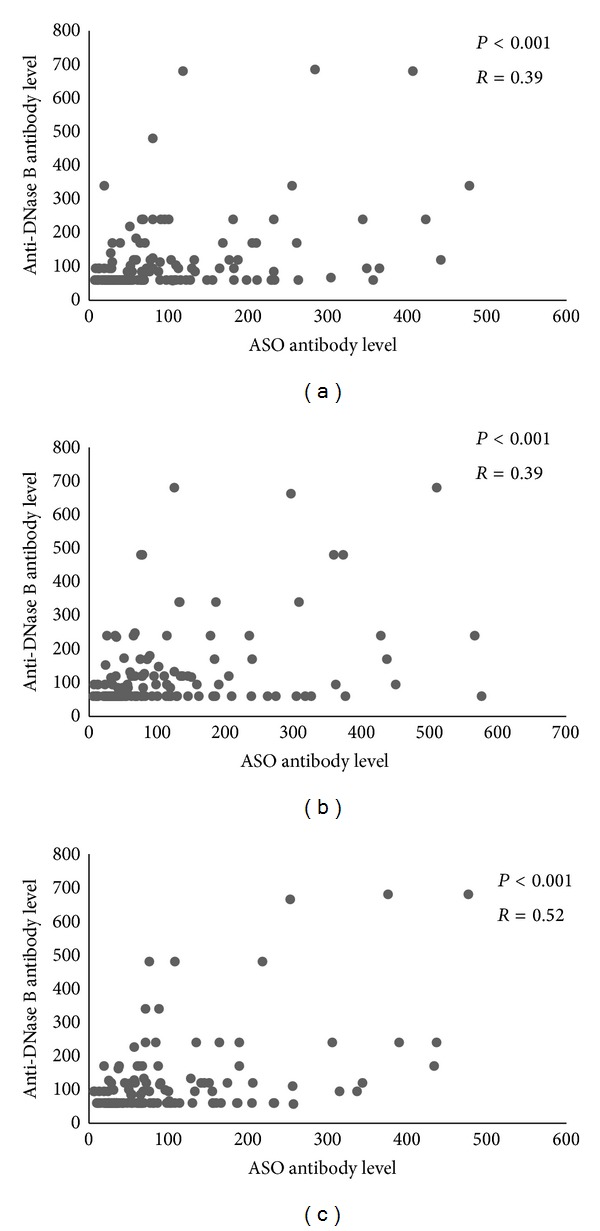
Correlation between ASO and anti-DNase B titers in pregnancy trimesters. Scatter plots showing correlation between values of ASO and anti-DNase-B antibodies in different pregnancy trimesters. Values less than laboratory lower limit for anti-DNase B detection were converted to the lower limit value of the assay. Correlation coefficients and Pearson correlation *P* values are shown on the plots: (a) Trimester 1, (b) Trimester 2, and (c) Trimester 3.

A comparison of the median change in ASO and anti-DNase B titers between pregnancy trimesters revealed a decrease in ASO titers throughout pregnancy while anti-DNase B titers did not change significantly between the second and third trimesters (Table 2). There was a positive correlation (but not strong) between values of ASO and anti-DNase B in all three trimesters (Figure 1).

The ULN values of ASO and anti-DNase B antibody titers in each trimester are shown in Table 3. A total of 13 subjects were excluded before calculation of ULN as described in the methods of the study. The ULN value for ASO declined with progression of pregnancy, but this value for anti-DNase B stayed constant at the time points measured during pregnancy.

The chance of having a positive throat culture for *β*-hemolytic streptococci at the time points sampled in the 3 trimesters (including carrier state and asymptomatic infections) was 34.1%. After excluding subjects with a previous history of tonsillectomy (32 patients), the incidence of having positive throat cultures was 40.4%. With regard to the serogroup of *β*-hemolytic streptococci in the cultured isolates, group C and group G strains together comprised 18.2% of patients while group F was detected in 13.5% of cases. The rate of colonization with GAS was 1.6%.  Table 4 summarizes throat culture results.

The proportion of smokers was significantly greater in subjects with positive throat culture for *β*-hemolytic streptococci (at any stage of pregnancy) compared with those with negative culture results (16.3% versus 3.6%, *P* = 0.013). Further, a greater proportion of subjects with positive cultures compared with negative cultures lived with a smoker (25.6% versus 10.8%, *P* = 0.032). In addition, subjects with at least one positive culture at any time during pregnancy lived in households in which the median number of children was greater than in subjects with negative cultures (median: 3 [IQR: 2.5–5] versus 2 [IQR: 1-2], *P* = 0.001).

Based on the ULN values, 28.6% of patients had recent streptococcal exposure. Subjects with laboratory evidence of recent strep exposure (defined as either ASO or anti-DNase B antibody titers of equal to, or more than, the ULN value during pregnancy), showed a trend toward an association with asthma when compared with unexposed individuals (33.3% versus 11.1%, *P* = 0.003 considering Bonferroni correction).

## 4. Discussion

In this study, we show for the first time, the rate of throat colonization with different groups of *β*-hemolytic streptococci among pregnant women in the US. Our data show a colonization rate of 34.1%, most of which caused by non-GAS *β*-hemolytic streptococci (groups G, C, and F). Strömberg et al. reported this incidence rate to be 19.4% among nonpregnant population, in a period of 2 years which is much lower than the reported rate in pregnant population [9].

Asymptomatic pharyngeal carriage of *β*-hemolytic streptococcus is a state of colonization of pharyngeal mucosa without the presence of an infection and the serological response to streptococcal antigen [18, 19]. As previously noted, some strains of groups C and G *β*-hemolytic streptococci cause infections similar to GAS [21] and can similarly induce rheumatogenic outcomes [16, 17]. A high colonization rate with these strains raises concerns about the possible emergence of drug-resistant rheumatogenic streptococci. Group F *β*-hemolytic streptococcus (*S. milleri* or* S. anginosus* group F) is generally considered a normal resident of healthy adult pharyngeal mucosa [22]. Nevertheless, it has been associated with purulent infections, particularly in those with underlying diseases [22, 23]. There were also reports of infections with* S. milleri* during pregnancy [24–26]. A high rate of colonization with group F streptococcus might also raise concerns about risk of* S. milleri*-induced infections in pregnant women, particularly in those with underlying medical conditions.

Our data show that the chance of colonization with *β*-hemolytic streptococcus is higher in pregnant women who are smokers and those with many children in the household compared with nonsmokers and/or households with few children. Evidence that smoking increases the rate of colonization with pathogenic respiratory agents has been previously reported [27, 28]. It has been proposed that smoking could increase bacterial adherence by inducing injury and alteration in innate immune response in oropharyngeal epithelial cells [29, 30].

In this study, we also defined previously unpublished ULN values of ASO and anti-DNase B antibodies in pregnant population. The ULN value of ASO in adult population has been reported in a range between 177 and 250 iu/mL among different geographical regions [31, 32]. Reported rates are greater than the titers we detected in our study for pregnant women.

Our results show an overall drop in ASO levels over the course of pregnancy, with anti-DNase B titers remaining almost stable during the last two-thirds of pregnancy. Many studies have emphasized the importance of reporting anti-DNase B titers to confirm recent streptococcal exposure [33, 34]. In a study by Blyth and Robertson [33], to detect streptococcal exposure in subjects with poststreptococcal nonsuppurative disease, 95% sensitivity was reported for the application of a combination of ASO and anti-DNase B tests, suggesting an increase in either of these tests should be used to detect recent streptococcus exposure. In our study, we used either of these two tests to detect recent exposure. We also showed a positive but not a strong linear correlation for levels of ASO and anti-DNase B; the correlation was found to be greater in the third trimester.

Pregnant women who have had recent exposure to *β*-hemolytic streptococcus were shown to have a trend toward having increased rates of asthma. A higher rate of GAS upper respiratory infection has been previously reported in asthmatic patients [35]. Whether the asthma is a risk factor for acquiring infection with *β*-hemolytic streptococcus strains or repetitive exposure to streptococcal sore throat (pharyngitis) could result in hyperreactivity of airways has not yet been clarified and requires further studies.

One of the limitations for our study is that we applied ULN values for ASO and anti-DNase B titers to detect recent streptococcus exposure. A drawback for applying the serology-based concept of ULN for detection of recent streptococcus exposure is the probability that, in a minority of the cases, titers might not increase enough to pass the ULN value after a recent exposure. Checking both ASO and anti-DNase B minimized this limitation, but it does not resolve it completely. Moreover, changes in ASO and anti-DNase B titer in pregnant women might be affected by serum volume changes during pregnancy. We were also unable to calculate, due to a limitation in sample size, an ULN 20% value of anti-DNase B for samples measured by nephelometric assay. Further, we measured throat colonization three times during pregnancy which may not indicate the exact incidence rate of colonization with *β*-hemolytic streptococci during pregnancy. Also, it was not possible for us to differentiate the carrier state with the “state of recent asymptomatic infection” for subjects with just one time isolation of streptococcus from throat (isolated at either the first or the third trimester). Lastly, cautions must be taken into account when comparing culture results from various studies because the methodology used in isolating streptococcal species in different laboratories might potentially make a difference in culture results.

## 5. Conclusion

In summary, we report for the first time that pregnancy is associated with a high rate of colonization with streptococcal group F and also streptococcal groups C and G and this colonization is more prevalent among smokers. In addition, we provided for the first time a reference value for ASO and anti-DNase B titers for pregnant women in the United States. Results of this study suggest that one other variable that must be considered at the time of interpreting ASO and anti-DNase B titer results is the occurrence of pregnancy. Our work shows that pregnant women act as a reservoir for spreading potentially immunogenic (groups C and G) and disease producing virulent (group F) strains of streptococci. How this colonization would affect the overall well-being of the mother, fetus, and households is an important question that warrants further investigations.

## Supplementary Material

The Supplementary File Attached to the Manuscript are Symbols (Units) Used in the Manuscript and Their Corresponding Meanings.

## Figures and Tables

**Table 1:** Demographic characteristics of pregnant women.

Characteristics	*N* (%)
Race	
White	103 (81.7)
African American	18 (14.3)
Asian and others	5 (4.0)
Age (years)—median (IQR)	31 (28–35)
Weight at visit 1 (kg)—median (IQR)	64 (59–78)
BMI at visit 1—median (IQR)	24.1 (21.3–28.6)

BMI: body mass index; IQR: interquartile range.

**Table 2:** Changes in ASO and anti-DNase B titers between pregnancy trimesters.

	Mean (SD)			Change T1 to T2 median (IQR)	Change T2 to T3 median (IQR)
	1st trimester	2nd trimester	3rd trimester		
ASO	112.0 (121.8)	105.9 (102.2)	102.8 (100.0)	−8 (−13, −1)^a^	−5 (−14, 0)^a^
Anti-DNase B					
Wampole kit	134.5 (133.8)	122.0 (117.5)	126.5 (125.4)	0 (0, 0)	0 (0, 0)
Nephelometer	132.0 (123.0)	114.6 (126.5)	117.9 (111.8)	0 (−45, 0)^b^	0 (−2.5, 1)

^a^ *P* < 0.001 (paired samples *t*-test).

^b^ *P* = 0.035 (paired samples *t*-test).

SD: standard deviation; IQR: interquartile range; ASO: antistreptolysin O.

**Table 3:** ULN values of the ASO and anti-DNase titers in pregnant women.

	1st trimester	2nd trimester	3rd trimester
ASO	164	157	156
Anti-DNase B^a^	170	170	170

^a^Just considering anti-DNase B titers measured by Wampole kit.

ULN: upper limit of normal; ASO: antistreptolysin O.

**Table 4:** Frequency of throat isolation of *β*-hemolytic streptococci in each pregnancy trimester and in total.

Lancefield group	Point prevalence *N* (%)			Incidence *N* (%)
	1st trimester	2nd trimester	3rd trimester	
A	1 (0.8)	—	1 (0.8)	2 (1.6)
B	2 (1.6)	2 (1.6)	2 (1.6)	3 (2.4)
C	5 (4.0)	6 (4.8)	7 (5.6)	15 (11.9)
F	5 (4.0)	9 (7.1)	8 (6.4)	17 (13.5)
G	5 (4.0)	2 (1.6)	6 (4.8)	9 (6.3)

Total	18 (14.3)^a^	19 (15.1)^a^	24 (19.0)^a^	43 (34.1)^a^

^a^Some subjects had more than one group isolated during the pregnancy.
