# Supplementary material for: Maternal β-Hemolytic Streptococcal Pharyngeal Exposure and Colonization in Pregnancy
Source: Infect Dis Obstet Gynecol. 2014 Aug 20;2014:639141. doi: 10.1155/2014/639141 (PMC4158157; doi:10.1155/2014/639141)
Supplement: Supplementary file 1 — The Supplementary File Attached to the Manuscript are Symbols (Units) Used in the Manuscript and Their Corresponding Meanings. [file 639141.f1.docx]

**Supplemental file**

Questionnaire to determine the mothers’ history of antecedent β-hemolytic streptococcal infection

Did you ever have any cough, sore throat, or other respiratory symptoms from the time period 3 months prior to this pregnancy until today?

- NO
- YES

Have you ever had any fever(s) over 100^o^ from the time period 3 months prior to this pregnancy until today?

- NO
- YES

Did you get strep throat or tonsillitis during this pregnancy?

- NO
- YES

Did you get strep throat or tonsillitis 3 months prior to this pregnancy?

- NO
- YES

Did you have contact with anyone who had strep throat or tonsillitis during this pregnancy?

- NO
- YES by whom? ________________________

Did you have contact with anyone who had strep throat or tonsillitis prior to this Pregnancy (i.e., from 3 months before until the estimated date of conception)?

- NO
- YES by whom? _________________________________

Are you currently on any antibiotics or have you taken any antibiotics during this pregnancy?

- NO
- YES

Have you ever had Rheumatic Fever?

- NO
- YES

When? ________________________________

Have you ever had Scarlet Fever?

- NO
- YES

When? ________________________________

In the past did you have repeated bouts of or a recurrent problem with strep throat or tonsillitis?

- NO
- YES

Did you have complications related to strep throat infection or tonsillitis?

- NO
- YES explain (skin rash___, tonsil removal__ other__)

How old were you when you first began having trouble with strep throat or tonsillitis?

- < 5 years
- 5-15 years
- 16 years and older

When you had strep throat or tonsillitis, did you ever get a rash?

- NO
- YES

When was the last time you had strep throat?

- < 1 year ago
- 1-3 years ago
- > 3 years ago

Have you had your tonsils removed?

- NO
- YES

If your tonsils were removed because of strep throat, did you continue to get strep throat after you had your tonsils removed?

- NO
- YES

How many are your biological children?

No. of Boys __________

No. of Girls __________

Have any of your children had recurrent strep throat or tonsillitis (i.e., more than once)?

- NO
- YES

Have any of your children been diagnosed as carriers of strep throat or tonsillitis?

- NO
- YES

Have any of your children had their tonsils removed?

- NO
- YES
